# Feature selection of gene expression data for Cancer classification using double RBF-kernels

**DOI:** 10.1186/s12859-018-2400-2

**Published:** 2018-10-29

**Authors:** Shenghui Liu, Chunrui Xu, Yusen Zhang, Jiaguo Liu, Bin Yu, Xiaoping Liu, Matthias Dehmer

**Affiliations:** 1 School of Mathematics and Statistics, Shandong University at Weihai, Weihai, 264209 China; 2 Genetics, Bioinformatics, and Computational Biology, Virginia Polytechnic Institute and State University, Blacksburg, VA USA; 3 College of Mathematics and Physics, Qingdao University of Science and Technology, Qingdao, 266061 China; 4 Institute for Intelligent Production, Faculty for Management, University of Applied Sciences Upper Austria, Steyr Campus, Steyr, Austria; 5 College of Computer and Control Engineering, Nankai University, Tianjin, 300071 China; 6 Department of Mechatronics and Biomedical Computer Science, UMIT, Hall in Tyrol, Austria

**Keywords:** Clustering, Gene expression, Cancer classification, Feature selection, Data mining

## Abstract

**Background:**

Using knowledge-based interpretation to analyze omics data can not only obtain essential information regarding various biological processes, but also reflect the current physiological status of cells and tissue. The major challenge to analyze gene expression data, with a large number of genes and small samples, is to extract disease-related information from a massive amount of redundant data and noise. Gene selection, eliminating redundant and irrelevant genes, has been a key step to address this problem.

**Results:**

The modified method was tested on four benchmark datasets with either two-class phenotypes or multiclass phenotypes, outperforming previous methods, with relatively higher accuracy, true positive rate, false positive rate and reduced runtime.

**Conclusions:**

This paper proposes an effective feature selection method, combining double RBF-kernels with weighted analysis, to extract feature genes from gene expression data, by exploring its nonlinear mapping ability.

**Electronic supplementary material:**

The online version of this article (10.1186/s12859-018-2400-2) contains supplementary material, which is available to authorized users.

## Background

Gene expression data can reflect gene activities and physiological status in a biological system at the transcriptome level. Gene expression data typically includes small samples but with high dimensions and noise [1]. A single gene chip or next generation sequencing technology can detect at least tens of thousands of genes for one sample, but when it comes to some diseases or biological processes, only a few groups of genes are related [2, 3]. Moreover, testing these redundant genes not only demands tremendous search space but also reduces the performance of data mining due to the overfitting problem. Thus, extracting the disease-mediated genes from the original gene expression data has been a major problem for medicine. Moreover, the identification of appropriate disease-related genes will allow the design of relevant therapeutic treatments [4, 5].

So far, several feature selection methods have been suggested to extract disease-mediated genes [6–8]. Zhou et al. [3] proposed a new measure, LS bound measure, to address numerous redundant genes. Several statistical theories (χ^2^et al.) and classic classifiers (Support Vector Machine et al.) have been used in feature selection [9]. In general, these methods can be divided into three categories: filter, wrapper and embedded methods [9, 10]. The filter method is based on the structural information of the dataset itself, which is independent of the classifier, and it selects a feature subset from the original dataset using a certain evaluation rule based on statistical methods [11]. The wrapper method [12] is based on the performance of the classifier to evaluate the significance of feature subsets, while the embedded method [13] combines the advantage of filter and wrapper methods, selecting feature genes using a pre-determined classification algorithm [14, 15]. Since the filter methods are independent of the classifier, the computational complexity of these methods is relatively low, hence, they are suitable for massive data processing [16]. Yet, wrapper methods can reach a higher accuracy, but they also have a higher risk of over-fitting.

Kernel methods have been one of the central methods in machine learning in recent years. They have widely been applied to the area of classification and regression. A kernel method has the capability of mapping the data (non-linearly) to a higher dimensional space [17]. Hence, by using the kernel method, the dimension of the observed data such as gene expression data can be significantly reduced, that is, the irrelevant genes can be filtered by kernel method, thus revealing the hidden inherent law in the biological system [18]. Characteristically, kernels have a great impact on learning and predictive results of machine learning methods [5, 19].

Although a great number of kernels exist and it is intricate to explain their distinctive characteristics, kernels used by feature extraction can be divided into two classes: global and local kernels, such as polynomial and radial basis function (RBF) kernels. The influence of different types of kernels on the interpolation and extrapolation capabilities has been investigated. In global kernels, data points far away from the test point have a profound effect on kernel values, while, by using local kernels, only those close to the test point have a great effect on kernel values. The polynomial kernel shows better extrapolation abilities at lower orders of the degrees, but requires higher orders of degrees for good interpolation, while the RBF-kernel has good interpolation abilities, but fails to provide longer range extrapolation [17, 20].

KBCGS [20] is a new filter method based on the RBF-kernel using weighted gene measures in clustering. This supervised learning algorithm applied global adaptive distance to avoid falling in local minima. The RBF kernel function has been proven useful when it comes to show a satisfactory global classification performance for gene selection. Yet, exploring this problem in depth definitely needs further research. A typical mixture kernel is to construct a convex combination of basis kernels. Based on the characteristics of the original kernel function, linear fusion of a local kernel function and a global kernel function can constitutes a new mixed kernel function. Several mixture kernels have been introduced in [21–23] to overcome limitations of single-kernel, which can enhance the interpretability of the decision, function and improve performance. Phienthrakul et al. proposed Multi-scale RBF Kernels in Support Vector Machines and demonstrated that the use of Multi-scale RBF Kernels could result in better performance than that of a single RBF on benchmarks [23].

In this paper, we modified KBCGS based on double RBF-kernels, and applied the proposed method to feature selection of gene expression. We introduced the double RBF-kernel to both SVM and KNN, and evaluated their performance in the area of gene selection. This mixture describes varying degrees of local and global characteristics of kernels only by choosing different values of γ_1_and γ_2_. We combined the double RBF-kernel with a weighted method to overcome the limitations of single and local kernel. As an application, we provided a feature extraction method which uses this kernel, applying our method to several benchmark datasets: diffuse large B-cell lymphoma (DCBL) [24], colon [2], lymphoma [1], gastric cancer [25], and mixed tumors [26] to evaluate its performance. The results demonstrate that this method allows better discrimination in gene selection. In addition, the method is superior when it comes to accuracy and efficiency if we compare this technique with traditional gene selection methods.

This paper provides a brief overview of the gene selection method for expression data analysis, then, the improved KBCGS method called DKBCGS (Double-kernel KBCGS), in which the two classification methods were used for the clustering analysis was compared to six popular gene selection methods. The last section of the paper provides a comprehensive evaluation of the proposed method using four benchmark gene expression datasets.

## Methods

Gene expression data with *l* genes and *n* samples can be represented by the following matrix:

1 $$ \mathrm{X}=\left[\begin{array}{ccc}{\mathrm{x}}_{11}& \cdots & {\mathrm{x}}_{1\mathrm{l}}\\ {}\vdots & \ddots & \vdots \\ {}{\mathrm{x}}_{\mathrm{n}1}& \cdots & {\mathrm{x}}_{\mathrm{n}\mathrm{l}}\end{array}\right] $$

*X*_*i*_is a row vector that represents the total gene expression levels of sample *i* and *x*_*ij*_ is the expression level of gene *j* of sample *i*.

### Cluster center

In this paper, we used Z-score to normalize the original data. The standard score *Z* used for a gene is as follows:

2 $$ Z=\frac{\left(x-\mu \right)}{\sigma } $$

where, *x* is the expression level of a gene in a sample, *μ* is the mean value of the gene across all samples, and *σ* is its standard deviation of the gene across all samples.

The cancer classification was formulated as a supervised learning problem, defining the cluster center as:

3 $$ {\mathrm{v}}_{\mathrm{ik}}=\frac{1}{\left|{C}_i\right|}\sum \limits_{X_j\in {C}_i}{x}_{jk} $$

In this equation, *I = 1, 2,…, C, j = 1,2,…,n, k = 1,2,…,l,* ***C***_***i***_ is the number of samples contained in class *C*_*i*_, respectively. Hence, *V*_*i*_ *=* [*v*_*i1*_*,…,v*_*il*_] is the cluster center of class *C*_*i*_.

### Double RBF-kernels

The kernel function acts as a similarity measure between samples in a feature space. A simple form of similarity measure is the dot product between two samples. The most frequently used kernel is a positive definite Gaussian kernel [27]. The classic Gaussian kernel on two samples *x* and x_*i*_, represented as feature vectors in an input space, is defined by:

4 $$ {\mathrm{K}}_{\mathrm{rbf}}\left(\mathrm{x},{\mathrm{x}}_{\mathrm{i}}\right)={\mathrm{e}}^{-{\upgamma}_1{\left\Vert \mathrm{x}-{\mathrm{x}}_{\mathrm{i}}\right\Vert}^2} $$

where, γ_1_ > 0 is a free parameter.

It is a positive definite kernel representing local features, therefore, it can also be used as the kernel function to weight genes for the gene selection method. Kernel methods have already been applied to many areas due to their effectiveness in feature selection and dimensionality reduction [27]. However, for the purposes of these methods, the focus is on creating a more general unified mixture kernel that has capabilities of both local and global kernels.

This work utilizes a double RBF-kernel as a similarity measure. The number choice of kernels could typically depend on the level of heterogeneity of the datasets. Increasing numbers of kernels helps to improve accuracy, but increase the computational cost. Therefore, we have to find a compromise between multiple kernels learning and double RBF-kernel learning, based on the performance and computational complexity. In most case, two RBF kernels are enough to handle most data with reasonable accuracy and computational cost. It should be emphasized that the proposed nonlinear kernel method is based on the combination of two RBF-kernels that has few limitations when calculating the distance among genes as follows:

5 $$ {\displaystyle \begin{array}{c}\kern1.5em {K}_{\gamma_1{\gamma}_2}\left(x,{x}_j\right)={ce}^{-{\gamma}_1{\left\Vert x-{x}_i\right\Vert}^2}+\left(1-\mathrm{c}\right){e}^{-{\gamma}_2{\left\Vert x-{x}_i\right\Vert}^2}\\ {}\kern1em \left({\gamma}_1>0,{\gamma}_2>0\right)\end{array}} $$

**Fig. 1:**
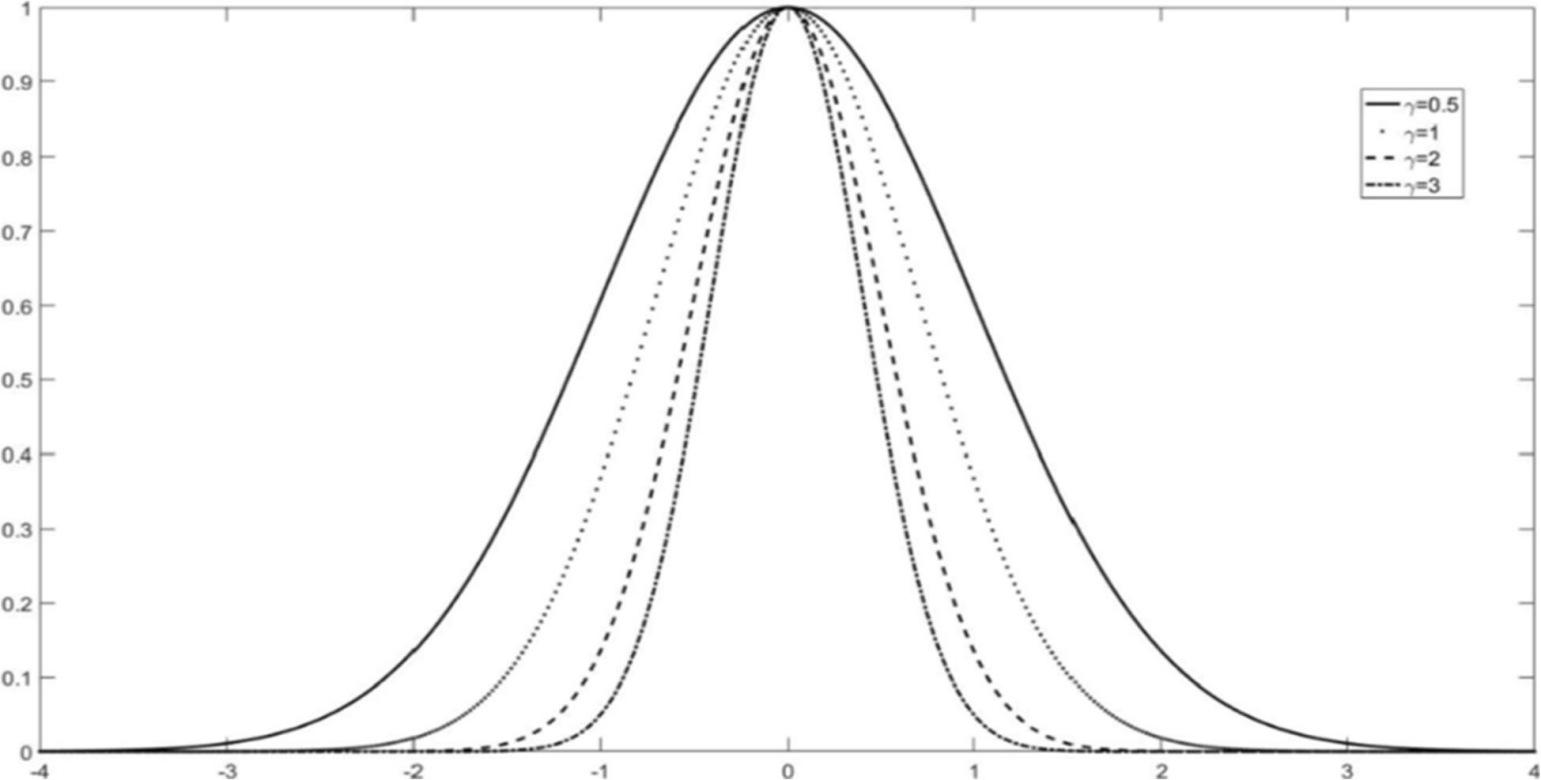
RBF kernel mapping with different γ_1_ for Eq. 13. Horizontal axis is ‖x − x_i_‖^2^. The vertical axis is K_rbf_(x, x_i_)

**Fig. 2:**
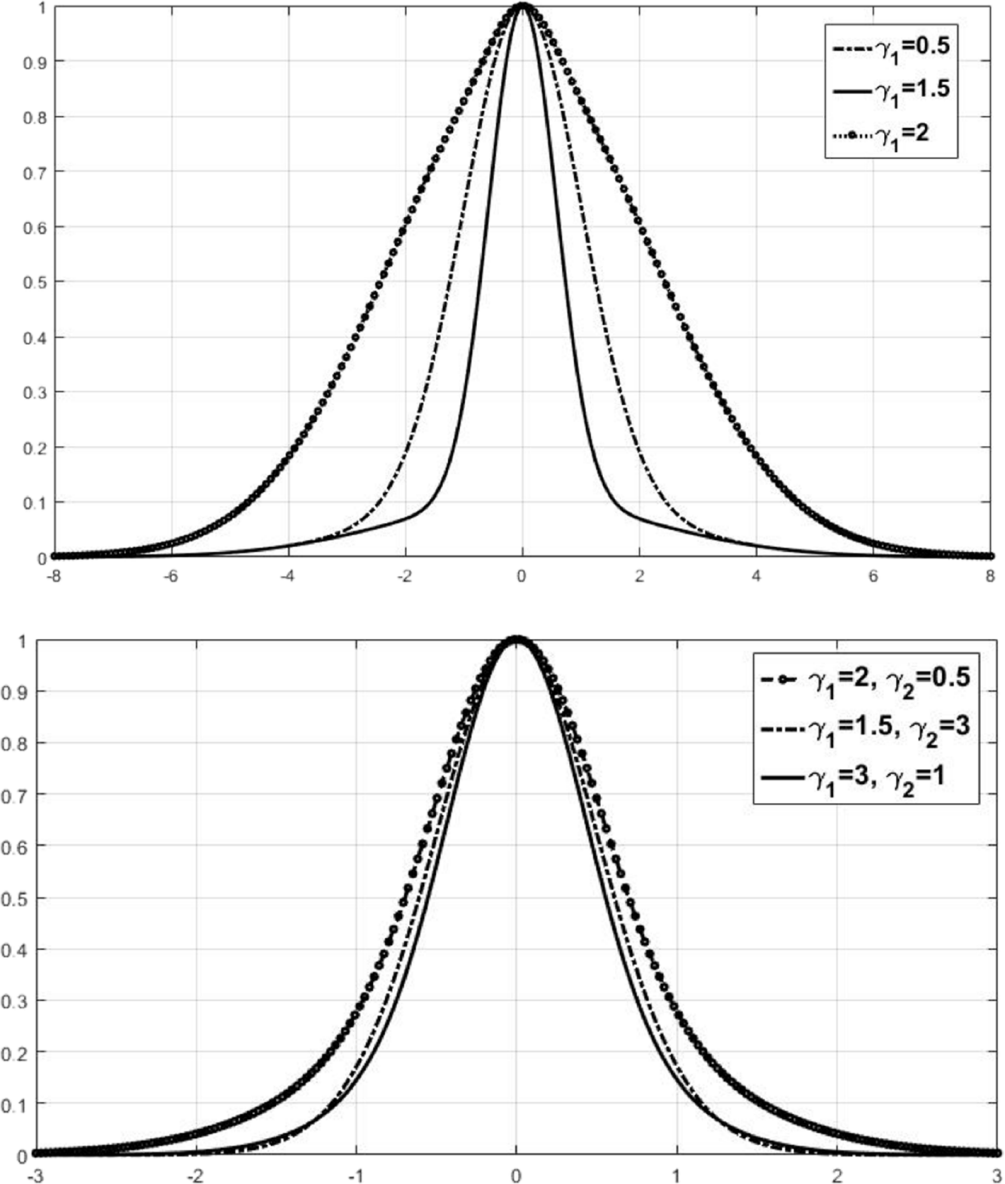
The mapping with different γ_1_ and γ_2_ for Eq. (5). The first figure is for γ_1_ only, and the second figure is for the combination of γ_2_ and γ_1_. The horizontal axis is given by ‖*x* − *x*_*i*_‖^2^ and the vertical axis is given by ${K}_{\gamma_1{\gamma}_2}\left(x,{x}_j\right)$

To further illustrate Eq. (5), the mapping relationships were plotted between the formula Eq. 5 and RBF-kernel by Figs. 1 and 2. Figures 1 and 2 clearly show the fat-tailed shape of the mapping changes with **γ**_**1**_ , **γ**_**2**_ and compared to the RBF mapping parameter *γ*_1_. Figure 2 shows changing parameters **γ**_**1**_ , **γ**_**2**_, the lower graph varies more slightly than the upper one. Therefore, the double-kernel can fit data better with less impact by outliers, indicating that the double-kernel has better flexibility than the single-kernel. The fat- tail characteristics make the double RBF kernels have better learning ability and better generalization ability than a RBF-kernel.

### Kernels as measures of similarity

Suppose Φ : X ⟶ F is a nonlinear mapping from the space X to a higher dimensional space F, By applying the mapping Φ, then the dot product ${\mathrm{x}}_{\mathrm{k}}^{\mathrm{T}}{\mathrm{x}}_{\mathrm{l}}$ in the input space X is mapped to Φ(x_k_)^T^Φ(x_l_) in the new feature space. The key idea in kernel algorithms is that the non-linear mapping Φ doesn’t need to be explicitly specified because each Mercer kernel can be expressed as:

6 $$ \mathrm{K}\left({x}_k,{x}_l\right)=\Phi {\left({x}_k\right)}^T\Phi \left({x}_l\right) $$

that is usually referred to as kernel trick [22]. Then, the Euclidean distances in F yields:

7 $$ {\displaystyle \begin{array}{c}{\left\Vert \Phi \left({x}_k\right)-\Phi \left({x}_l\right)\right\Vert}^2={\left(\Phi \left({x}_k\right)-\Phi \left({x}_l\right)\right)}^T\Big(\Phi \left({x}_k\right)-\Phi \left({x}_l\right)\\ {}\kern4.559998em =\mathrm{K}\left({x}_k,{x}_k\right)-2\mathrm{K}\left({x}_k,{x}_l\right)+\mathrm{K}\left({x}_l,{x}_l\right)\end{array}} $$

Then, a dissimilarity function between an sample and a cluster centroid could be defined as:

8 $$ {\displaystyle \begin{array}{c}{\phi}^2\left({x}_j,{v}_i\right)={\sum}_{k=1}^l{\left\Vert \Phi \left({x}_{jk}\right)-\Phi \left({v}_{ik}\right)\right\Vert}^2\\ {}={\sum}_{k=1}^l\left(\mathrm{K}\left({x}_{jk},{x}_{jk}\right)-2\mathrm{K}\left({x}_{jk},{v}_{ik}\right)+\mathrm{K}\left({v}_{ik},{v}_{ik}\right)\right)\end{array}} $$

### Gene ranking and selection

The most used gene selection methods belong to the so-called filter approach. Filter-based feature ranking methods rank genes independently without any learning algorithm. Feature ranking consists of weighting each feature according to a particular method, then selecting genes based on their weights.

In this paper, our method DKBCGS is based on a KBCGS method improved to achieve higher accuracy and converge faster.

The KBCGS method adopted global distance, assigning different weights to different genes. The clustering objective function is given by:

9 $$ {\displaystyle \begin{array}{c}\mathrm{J}={\sum}_{\mathrm{i}=1}^{\mathrm{C}}{\sum}_{{\mathrm{x}}_{\mathrm{j}}\in {\mathrm{C}}_{\mathrm{i}}}{\upphi}^2\left({\mathrm{X}}_{\mathrm{j}},{\mathrm{V}}_{\mathrm{i}}\right)+\updelta {\sum}_{\mathrm{k}=1}^{\mathrm{l}}{\mathrm{W}}_{\mathrm{k}}^2\\ {}={\sum}_{\mathrm{i}=1}^{\mathrm{C}}{\sum}_{{\mathrm{x}}_{\mathrm{j}}\in {\mathrm{C}}_{\mathrm{i}}}{\sum}_{\mathrm{k}=1}^{\mathrm{l}}{\mathrm{W}}_{\mathrm{k}}{\left\Vert \Phi \left({\mathrm{X}}_{\mathrm{j}\mathrm{k}}\right)-\Phi \left({\mathrm{V}}_{\mathrm{i}\mathrm{k}}\right)\right\Vert}^2+\updelta {\sum}_{\mathrm{k}=1}^{\mathrm{l}}{\mathrm{W}}_{\mathrm{k}}^2\end{array}} $$

where w = (w_1_, w_2_,...,w_l_) are the weight of genes.

10 $$ \Big\{{\displaystyle \begin{array}{c}{\mathrm{w}}_{\mathrm{k}}\in \left[0,1\right],\mathrm{k}=1,2,\dots, 1\\ {}{\sum}_{\mathrm{k}=1}^{\mathrm{l}}{\mathrm{w}}_{\mathrm{k}}=1\end{array}}\operatorname{} $$

As shown in Eq. (1), the first part is the sum of weighted dissimilarity distance among samples and the cluster they belong to evaluated by the kernel method. This part will reach its minimum value only when there is one gene that is completely relevant and the others are irrelevant. The second part is the sum of squared weights of genes, which will only reach its minimum value when all genes are equally weighted. Therefore, by combining these two parts, the optimal gene weights are obtained, then the feature genes can be selected.

To minimize J with respect to the restriction Eq. (10), the Lagrange multipliers methods were applied as follows:

11 $$ \mathrm{J}\left({\mathrm{w}}_{\mathrm{k}},\uplambda \right)={\sum}_{\mathrm{i}=1}^{\mathrm{C}}{\sum}_{{\mathrm{x}}_{\mathrm{j}}\in {\mathrm{C}}_{\mathrm{i}}}{\upphi}^2\left({\mathrm{x}}_{\mathrm{j}},{\mathrm{v}}_{\mathrm{i}}\right)+\updelta {\sum}_{\mathrm{k}=1}^{\mathrm{l}}{\mathrm{w}}_{\mathrm{k}}^2-\uplambda \left({\sum}_{\mathrm{k}=1}^{\mathrm{l}}{\mathrm{w}}_{\mathrm{k}}-1\right) $$

So, the partial derivative of J(w_k_, λ) is given by:

12 $$ \left\{\begin{array}{l}\frac{\mathrm{\partial J}\left({\mathrm{w}}_{\mathrm{k}},\uplambda \right)}{\mathrm{\partial \uplambda }}={\sum}_{\mathrm{k}=1}^{\mathrm{l}}{\mathrm{w}}_{\mathrm{k}}-1\\ {}\frac{\mathrm{\partial J}\left({\mathrm{w}}_{\mathrm{k}},\uplambda \right)}{\partial {\mathrm{w}}_{\mathrm{k}}}={\sum}_{\mathrm{i}=1}^{\mathrm{C}}{\sum}_{{\mathrm{x}}_{\mathrm{j}}\in {\mathrm{C}}_{\mathrm{i}}}{\left\Vert \Phi \left({\mathrm{x}}_{\mathrm{j}\mathrm{k}}\right)-\Phi \left({\mathrm{v}}_{\mathrm{i}\mathrm{k}}\right)\right\Vert}^2+2{\updelta \mathrm{w}}_{\mathrm{k}}-\uplambda \end{array}\right. $$

The J(w_k_, λ) reaches its minimum when the value of the partial derivative is zero. So, w is calculated as follows:

13 $$ {\mathrm{w}}_{\mathrm{k}}=\frac{1}{\mathrm{l}}+\frac{1}{2\updelta}{\sum}_{\mathrm{i}=1}^{\mathrm{C}}{\sum}_{{\mathrm{x}}_{\mathrm{j}}\in {\mathrm{C}}_{\mathrm{i}}}\left(\frac{\sum_{\mathrm{i}=1}^{\mathrm{C}}{\sum}_{{\mathrm{x}}_{\mathrm{j}}\in {\mathrm{C}}_{\mathrm{i}}}{\left\Vert \Phi \left({\mathrm{x}}_{\mathrm{j}\mathrm{k}}\right)-\Phi \left({\mathrm{v}}_{\mathrm{i}\mathrm{k}}\right)\right\Vert}^2}{1}-{\left\Vert \Phi \left({\mathrm{x}}_{\mathrm{j}\mathrm{k}}\right)-\Phi \left({\mathrm{v}}_{\mathrm{i}\mathrm{k}}\right)\right\Vert}^2\right) $$

Based on Eq. (13), the KBCGS method chooses $\frac{1}{\mathrm{l}}$ as the initial weight of w_k_. In the second part of Eq. (9), the choice of δ is quite important since it represents the distance of genes. The value of δ should ensure that both parts are of the same order of magnitude, so according to SCAD algorithm [28], the δ is calculated iteratively as follows:

14 $$ {\updelta}^{\left(\mathrm{t}\right)}=\upalpha \frac{\sum_{\mathrm{i}=1}^{\mathrm{C}}{\sum}_{{\mathrm{x}}_{\mathrm{j}}\in {\mathrm{C}}_{\mathrm{i}}}{\sum}_{\mathrm{k}=1}^{\mathrm{l}}{\mathrm{w}}_{\mathrm{k}}^{\left(\mathrm{t}-1\right)}{\left\Vert \Phi \left({\mathrm{x}}_{\mathrm{j}\mathrm{k}}\right)-\Phi \left({\mathrm{v}}_{\mathrm{i}\mathrm{k}}\right)\right\Vert}^2}{\sum_{\mathrm{k}=1}^{\mathrm{l}}{\left({\mathrm{w}}_{\mathrm{k}}^{\left(\mathrm{t}-1\right)}\right)}^2} $$

Where α is a constant which influences the value of δ, with a default value of 0.0S5. The Gaussian kernel is employed in this algorithm:

15 $$ {\mathrm{K}}_{\mathrm{rbf}}\left(\mathrm{x},{\mathrm{x}}_{\mathrm{i}}\right)={\mathrm{e}}^{-{\upgamma}_1{\left\Vert \mathrm{x}-{\mathrm{x}}_{\mathrm{i}}\right\Vert}^2} $$

Where, γ_1_ > 0 is a free parameter and the distance can be expressed as:

16 $$ \left\Vert \Phi \left({\mathrm{x}}_{\mathrm{jk}}\right)\right.-{\left.\Phi \left({\mathrm{v}}_{\mathrm{ik}}\right)\right\Vert}^2=2\Big(1-\mathrm{K}\left({\mathrm{x}}_{\mathrm{jk}},{\mathrm{v}}_{\mathrm{ik}}\right) $$

The max number of iteration is 100, and θ = 10^− 6^.

The features of the improved method are outlined below. Similar to KBCGS algorithm [20], the clustering objective function is defined:

$$ J={\sum}_{i= 1}^C{\sum}_{x_j\in {C}_i}{\phi}^2\left({x}_j,{v}_i\right)+\delta {\sum}_{k= 1}^l{w}_k^2 $$

where *w = (w*_*1*_*, w*_*2*_*,...,w*_*l*_*)* are the weight of genes.

The DKBCGS method calculates δ iteratively according to Chen’s approach [20], however, it is improved the iterative method to calculate w by deriving the following formula:

17 $$ {\delta}^{(t)}=\left|\frac{\sum_{i=1}^C{\sum}_{xj\in {C}_i}{\sum}_{k=1}^l{w}_k^{\left(t-1\right)}{\left\Vert \Phi \left({x}_{jk}\right)-\Phi \left({v}_{ik}\right)\right\Vert}^2}{\sum_{k=1}^1{\left({w}_k^{\left(t-1\right)}\right)}^2}\right| $$

and instead of Gaussian kernel, the double RBF-kernel is used as mentioned in Eq. (5).

The initial value of δ in Eq. (13) is important in our algorithm since it reflects the importance of the second term relative to the first term. If δ is too small, the only one feature in cluster i will be relevant and assigned a weight of one. All other feature will be assigned zero weights. On the other hand, if δ is too large, then all feature in cluster I will be relevant, and assigned equal weights of 1/n. The values of δ should be chosen such that both terms are of same order of magnitude. In all examples described in this paper, we compute δ iteratively using Eq. (17) as SCAD method, see [28].

Through improving the iteration method, we achieve less iteration, therefore an improvement toward convergence compared to the KBCGS method. As previously mentioned, gene expression datasets are often linearly non-separable, so choosing an appropriate nonlinear kernel to map the data to a higher dimensional space has been proven efficient.

### Implementation

The algorithm can be stated using the following pseudocode:

Input: Gene expression dataset X and class label vector y;

Output: weights vector w of genes;

Use Z-score to normalize the original data X;

Use Eq. (3) to calculate the cluster center of different class of genes in the input space, respectively;

Use Eq. (8) to calculate the dissimilarity between the genes and their cluster center of class;

Initial value: w_0_ =$$ \frac{1}{\mathrm{l}} $$;

Repeat:

Use Eq. (14) to find the (*t + 1)*th distance parameter δ^(t + 1)^;

Use Eq. (13) to calculate (*t + 1)*th weights w^(t + 1)^ of genes;

Use Eq. (11) to calculate (*t + 1)*th objective function J^(t + 1)^;

Until: J^(t + 1)^-J^(t)^ < θ.

Return w^(t + 1)^.

We constructed SVM and KNN classifiers for each dataset. These methods have been introduced in the Additional file 2. A 10-fold cross validation was used as the validation strategy to reduce the error and obtain classification accuracy.

**Fig. 3:**
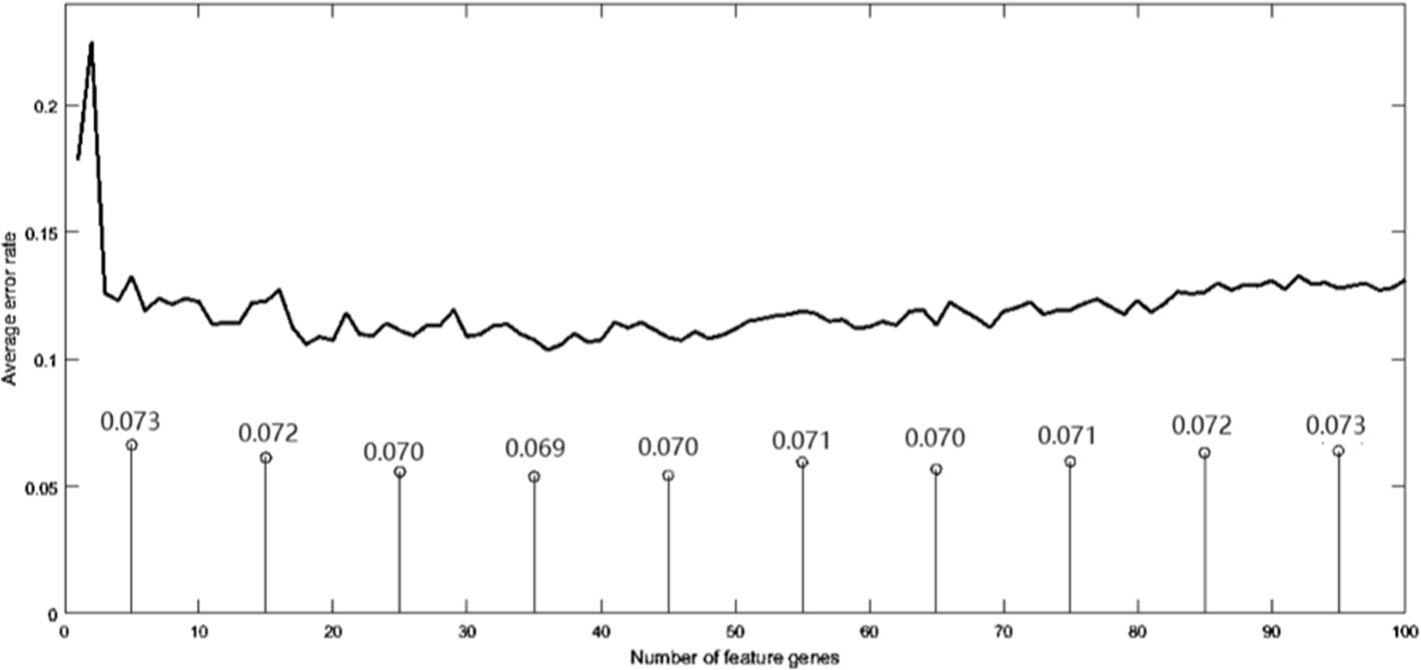
Average error rate versus different number of selected feature genes

The whole experiment was performed using MATLAB. To determine the value of hyperparameters, we use the grid search method. Figure 3 shows the change of in the average error rate with the change in the number of selected feature genes by employing DKBCGS. It is obvious that there is a great improvement in the results when the selected feature genes number increases from 1 to 20. In order to identify the optimal performance of all datasets, the number was restricted from 1 to 50.

## Results

To validate the performance of DKBCGS method, it was compared with some commonly used filter-based feature ranking methods namely*χ*^2^-Statistic, Maximum relevance and minimum redundancy (MRMR), Relief-F, Information Gain and Fisher Score. These methods have been introduced in the Additional file 1. Also, the improved approach was compared with KBCGS [20].

### Dataset description

The four datasets used as benchmark examples in this work are shown in Table 1. The specifics of these datasets are outlined in the Additional file 3.

**Table 1:** Summary of the four gene expression datasets

	Samples	Classes	Genes	References
DLBCL	77	2	7129	Shipp et al. [24]
Gastric cancer	40	2	1519	Boussioutas et al. [25]
Multi-cancer	152	5	65,522	Yuan et al. [26]
Lymphoma	62	3	4026	Alizadeh et al. [1]

## Discussion

By using the two-class datasets, the performance of proposed method, in comparison to the other six methods, was evaluated by calculating the accuracy (ACC), the true positive rate (TPR) and the true negative rate (TNR).

Table 2 and Table S1 shows the results of the two-class datasets. These results indicate that the proposed method has high accuracy and short runtime in both the SVM and KNN classifier, while MRMR also performs well in the KNN classifier. Fig. S1 tell us that the expression of the characteristic genes selected by the proposed algorithm has significant differences in the expression level of normal/diseased samples.

Gene-set enrichment analysis is used to identify coherent gene-sets. Fig. 5 show us that the genes (dataset: colon cancer), selected by DKBCGS, enriched in strongly connected gene-gene interaction networks and in highly significant biological processes. Furthermore, the significant difference between the expression profiles for the top-ranked genes selected by DKBCGS in the form of a color map in Fig. 6 (a) and the expression profiles for eight genes chosen randomly from the base is presented in Fig. 6 (b) confirms the good performance of the proposed selection procedure.

### Classification accuracy

18 $$ Accuracy=\frac{TP+ TN}{TP+ FP+ TN+ FN}\kern0.6em 0\leqslant ACC\leqslant 1 $$

TP, TN, FP, FN are the True Negatives, True Positives, False Negatives and False Positives, respectively.

As the number of positive samples and negative samples using the two-class datasets are not equal, the true positive rate (TPR) and the true negative rate (TNR) were used as another strategy for measuring the performance, considering both the precision and the recall of the experiment under test. Precision represents the number of correct positive results divided by the number of all positive results. Recall is the number of correct positive results divided by the number of positive results that should have been returned. Therefore, the TPR and false positive rate (FPR) are calculated as follows:

### True positive rate

19 $$ \mathrm{TPR}=\frac{TP}{TP+ FN} $$

### True negative rate

20 $$ \mathrm{TNR}=\frac{TN}{FP+ TN} $$

**Fig. 4:**
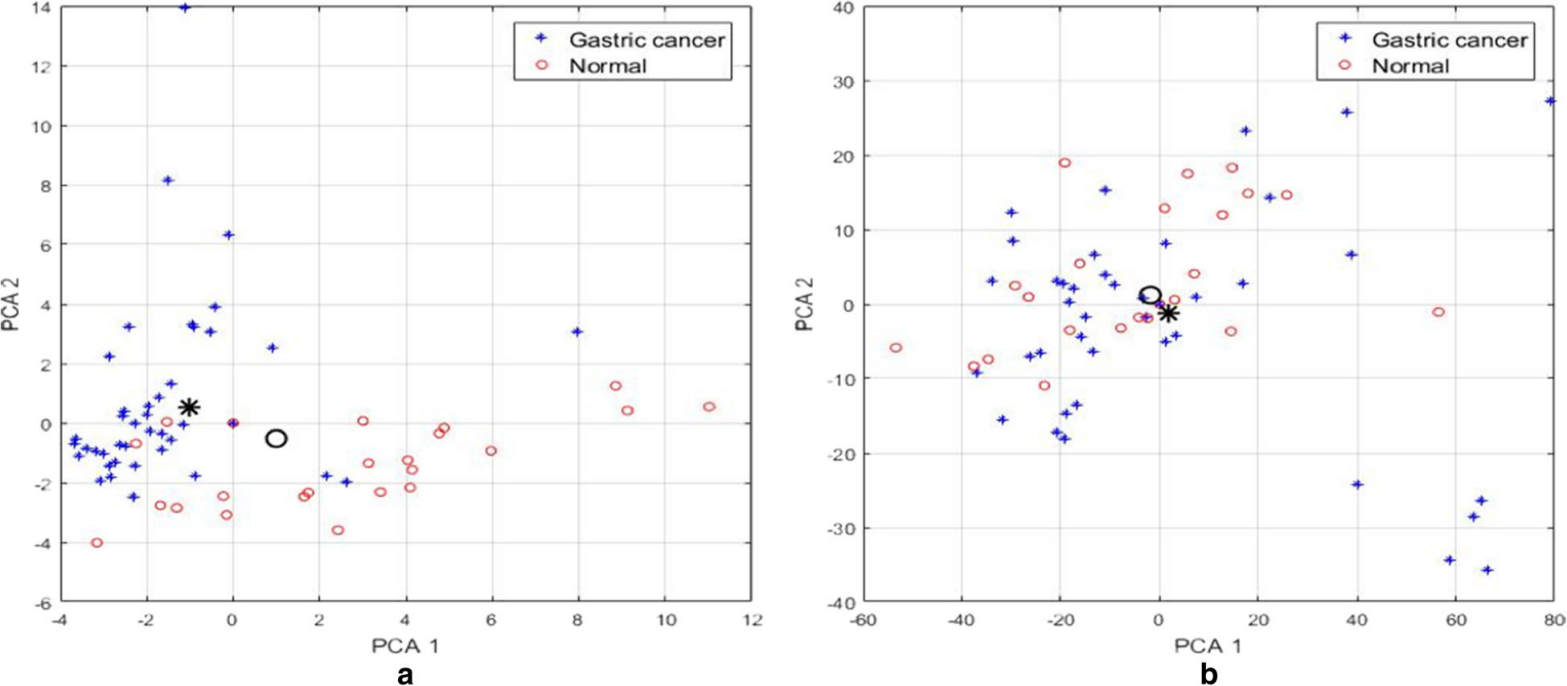
The distribution of the two-class samples mapped on the two most important principal components at representation of vectors x by 50 most significant genes (**a**) and at application of all genes (**b**). The horizontal axis is the first principal component and the vertical axis is the second principal component. Black marks represent different categories of the centers

Table 2 shows the results of the two-class datasets. The runtime of DKBCGS, being less than 0.1 s, is much shorter than others, except for runtime of MRMR-SVM in the DLBCL dataset, that is, the proposed double-kernel model can efficiently reduce computation complexity. Regarding accuracy, the proposed method also performs well, reaching 100% in SVM classifier and slightly less than that of MRMR in KNN classifier. Taken together, these results indicate that the proposed method has high accuracy and short runtime in both the SVM and KNN classifier, while MRMR also performs well in the KNN classifier. Also, the average ROC (Receiver Operating Characteristic) curve was plotted for further evaluation in Fig. 4. A further comparison with KBCGS in four datasets, calculating average results of KNN and SVM, is shown in Additional file 4: Table S1. The results clearly demonstrate that the improved approach DKBCGS performs better in both runtime and accuracy.

**Table 2:** Performance of gene feature selection methods with KNN classifier (high) and SVM classifier (low) in two-class datasets

Dataset: Gastric cancer								
	DKBCGS	GINI	Χ^2^-Statistic	Info.Gain	KW	RF	MRMR	KBCGS
ACC	0.9821	0.9664	0.9875	0.9779	0.9038	0.9548	**0.9986**	0.9716
TNR	1.0000	0.9500	0.9367	1.0000	0.9500	0.9800	1.0000	0.9755
TPR	0.9818	0.9677	0.9969	0.9759	0.8771	0.9498	1.0000	0.9826
TIME(s)	**0.0846**	0.7349	1.4736	0.7542	9.7452	4.2604	0.9007	0.6518
Dataset: DLBCL								
	DKBCGS	GINI	Χ^2^-Statistic	Info.Gain	KW	RF	MRMR	KBCGS
ACC	**0.9833**	0.9615	0.9865	0.9712	0.9123	0.9245	0.9341	0.9795
TNR	0.9943	0.9456	0.9422	0.9854	0.9457	0.9456	0.9654	1.0000
TPR	0.9863	0.9513	0.9645	0.9541	0.9024	0.9234	0.9432	0.9712
TIME(s)	0.1215	0.2257	0.1954	0.1857	0.1678	0.5111	**0.0931**	0.2148
Dataset: Gastric cancer								
	DKBCGS	GINI	Χ^2^-Statistic	Info.Gain	KW	RF	MRMR	KBCGS
ACC	**1.0000**	0.9768	0.9855	0.9623	0.9168	0.973	0.9988	0.9822
TNR	1.0000	0.9611	0.95	0.9158	0.9316	0.9433	1.0000	1.0000
TPR	1.0000	0.9929	0.9971	0.9776	0.9121	0.9827	1.0000	0.9755
TIME(s)	**0.0846**	0.7349	1.4736	0.7542	9.7452	4.2604	0.9007	0.7418
Dataset: DLBCL								
	DKBCGS	GINI	Χ^2^-Statistic	Info.Gain	KW	RF	MRMR	KBCGS
ACC	**1.0000**	**1**	0.9975	1.0000	0.9975	0.9750	0.9975	0.9845
TNR	1.0000	1.0000	1.0000	1.0000	0.9683	0.9733	0.9571	0.9579
TPR	1.0000	1.0000	1.0000	1.0000	0.8383	0.9437	0.9917	0.9931
TIME(s)	0.1215	0.2257	0.1954	0.1857	1.6478	0.5111	**0.0931**	0.2148

Regarding the gastric cancer dataset, we have mapped the multidimensional observations into 2-dimensional space formed by the two most important principal components.

**Fig. 5:**
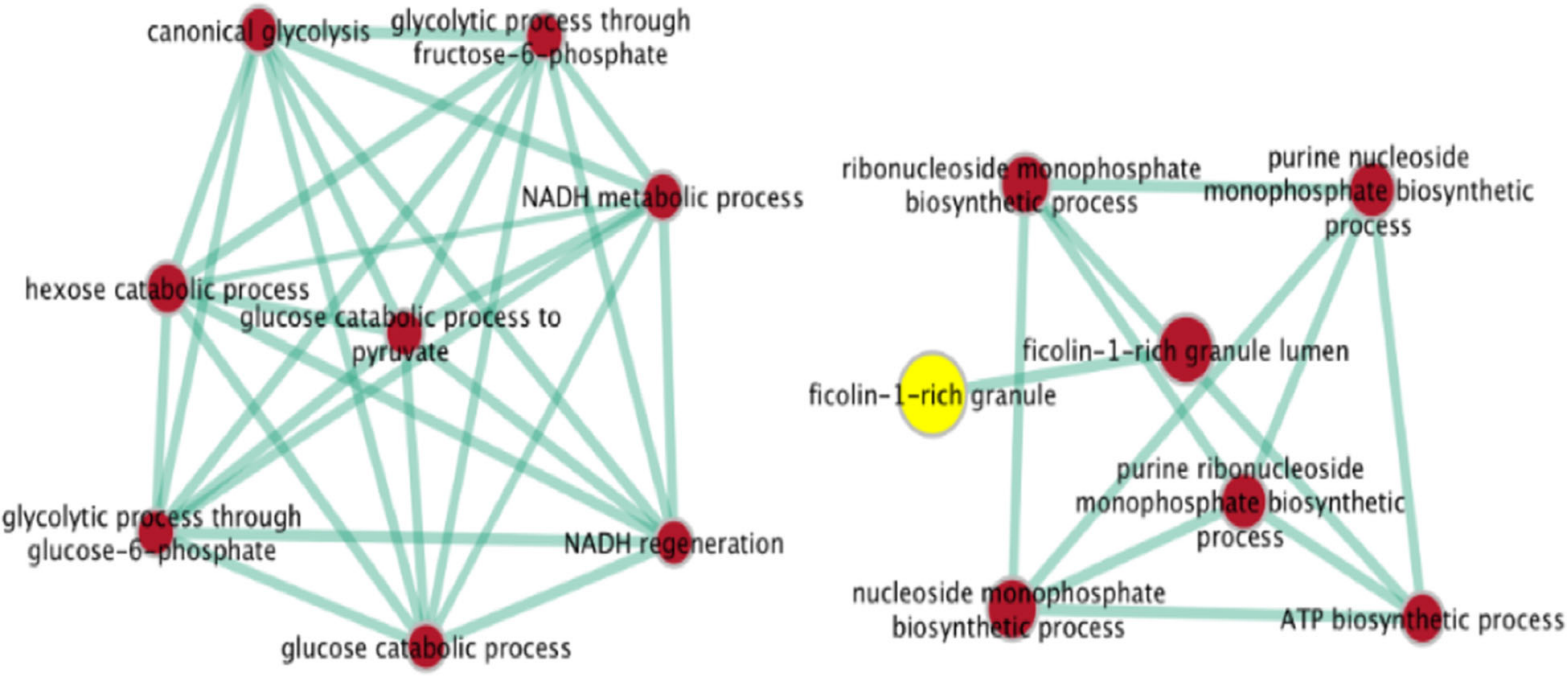
GO Enrichment Mapping the cluster-specific genes for the DLBCL dataset (P-value < 0.001). We firstly identified significant GO terms on the g: profiler web interface. Then we used the enrichment map plug-in in Cytoscape [29] to visualize these significant GO terms. Each node represents a GO term and each edge represents the degree of gene overlap (Jaccard similarity) that exists between two gene sets corresponding to the two GO terms

Two cases have been investigated. The first approach deals with using the original vectors only containing 50 genes selected by the fusion procedure. Fig. 5(a) depicts this case in which only the best representative genes in the vector x are used. For comparison, the Principal component analysis (PCA) was repeated for the full-size original 2000 element vectors containing all genes. The graphical results of the sample distribution are presented in Fig. 5(b). Large bold symbols of the circle and x represent the centroids of the data belong to two classes.

Furthermore, the first fifty top-ranked gene expression levels were analyzed in the gastric cancer dataset using the various methods as shown in Additional file 5: Figure S1. It can be clearly seen that the expression of the characteristic genes selected by the proposed algorithm has significant differences in the expression level of normal/diseased samples, therefore has some research value.

### Gene-set enrichment analysis

**Fig. 6:**
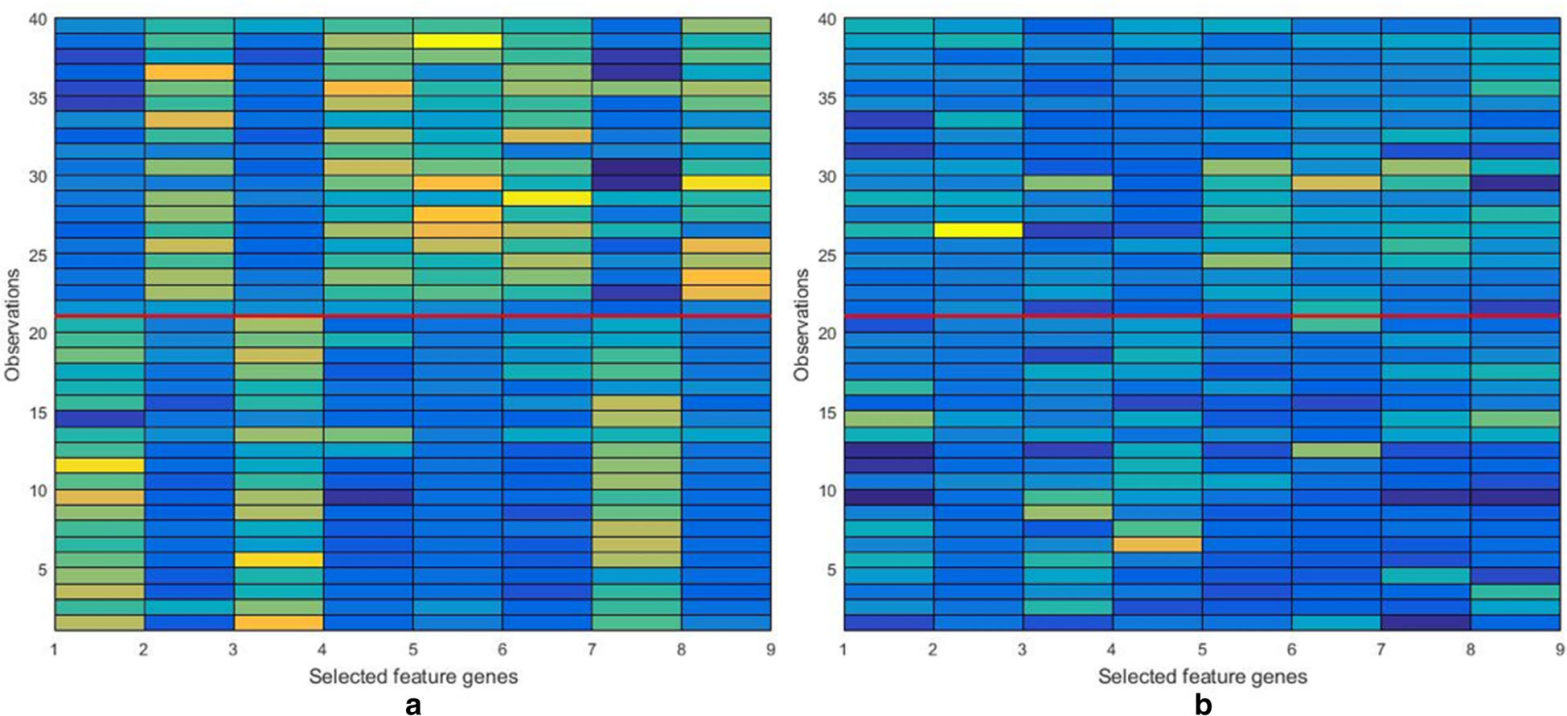
The colormap of the expression profiles for nine most significant genes selected by DKBCGS (**a**) and for 9 randomly chosen genes (**b**). The red line distinguishes between cancer samples and normal samples

Gene-set enrichment analysis is useful to identify coherent gene-sets, such as pathways, that are statistically overrepresented in a given gene list. Ideally, the number of resulting sets is smaller than the number of genes in the list, thus simplifying interpretation. However, the increasing number and redundancy of gene-sets used by many current enrichment analysis resources work against this ideal. Gene-sets are organized in a network, where each set is a node and links the representative gene overlap between sets [26]. So, as to dataset DLBCL, the genes selected by DKBCGS enriched in strongly connected gene-gene interaction networks and in highly significant biological processes (Fig. 6).

**Fig. 7:**
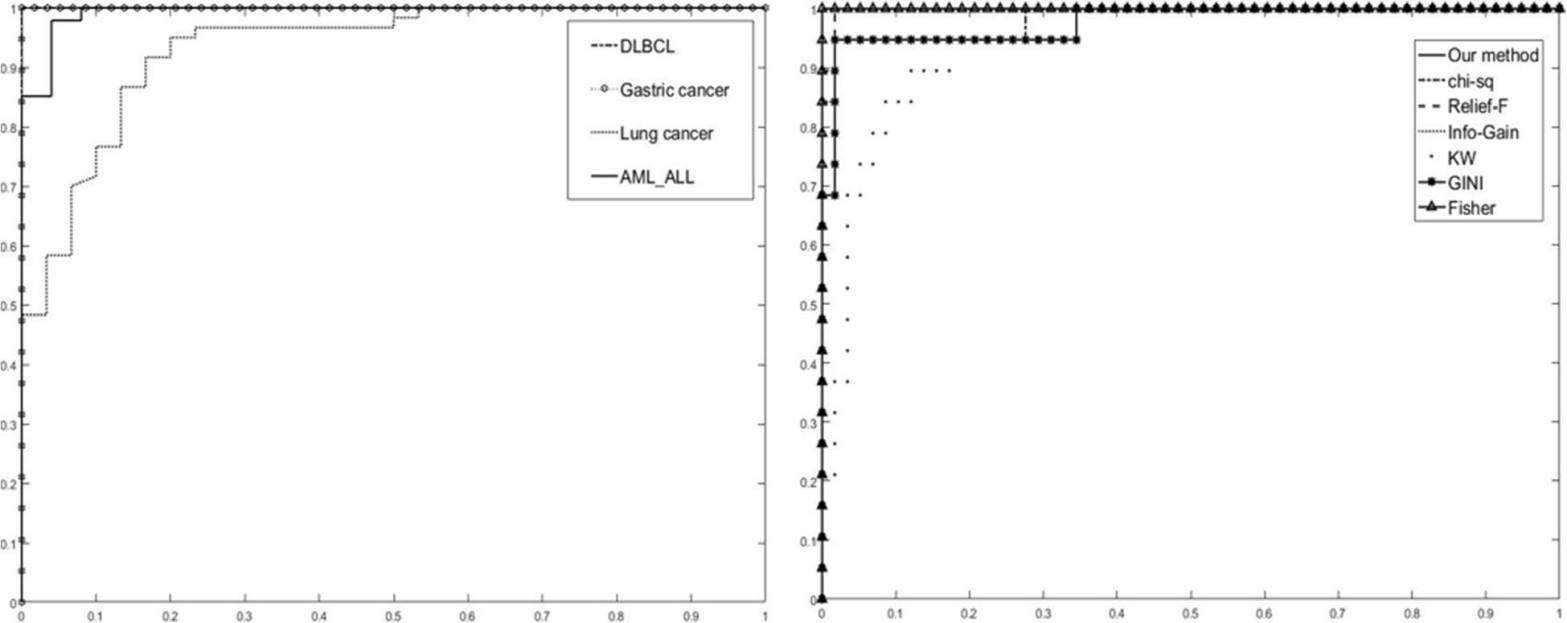
The ROC curve of two-class datasets, (left) ROC curve in different datasets and (right) shows the performance of different methods in DLBCL dataset. The horizontal axis is the false positive rate; the vertical axis is the true positive rate

To illustrate the results in a graphical form, the expression levels of the selected genes (dataset: colon cancer) are presented in Fig. 7(a). This figure shows the image of the expression profiles for the top-ranked genes selected by DKBCGS in the form of a colormap. The vertical axis represents observations and the horizontal axis represents the genes arranged according to their importance. There is a visible border between the cancer group and the normal group. For comparison purposes, the image of the expression profiles for eight genes chosen randomly from the base is presented in Fig. 7(b). There is a significant difference between both images, which confirms the good performance of the proposed selection procedure.

**Fig. 8:**
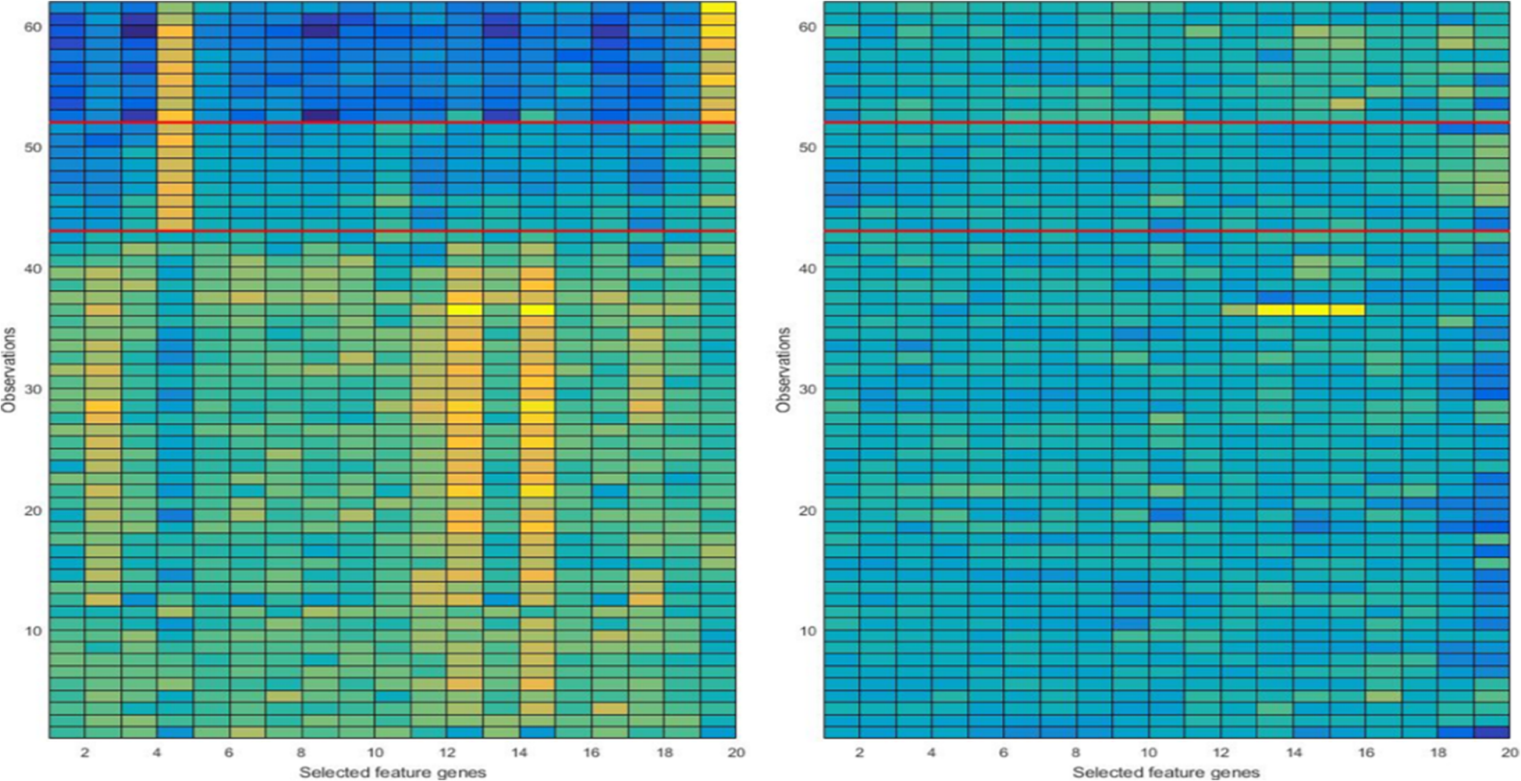
The colormap of the expression profiles for 20 most significant genes selected by the proposed method (left) and for 20 randomly chosen genes (right). The red line distinguishes between different classes

 Both Table 3 and Table S2 show the results of the multiclass datasets. Both tables clearly show that the KBCGS can reduce runtime with high accuracy in other multiclass datasets. When using the lung cancer gene expression data, there is a substantial improvement in the accuracy of the classification using the double RBF-kernel algorithm for each of the feature subsets, which demonstrates that the KBCGS method can select the appropriate genes efficiently compared to other methods. For lung cancers, the feature genes selected by the double RBF-kernel algorithm also result in a higher accuracy. It not only improves the accuracy of the classification of gene expression data, but also identifies informative genes that are responsible for causing diseases. Therefore, the double RBF-kernel method is better than the Χ2-Statistics, MRMR, Relief-F, Information Gain, and Kruskal-Wallis test. Also, the significant difference between the expression profiles for the top-ranked genes (dataset: Lymphoma) selected by DKBCGS in the form of a color map in Fig. 8 (a) and the expression profiles for 20 genes chosen randomly from the base is presented in Fig. 8 (b) demonstrates the good performance of the proposed selection procedure.

### Comparison of multiclass datasets

For the multiclass datasets, the performance of all methods was evaluated by computing accuracy (ACC) and run time (Time). The results are shown in Table 3. Also, further comparisons were made with KBCGS in other multiclass datasets, see Additional file 4: Table S2. Both tables clearly show that the proposed method can reduce runtime with high accuracy.

**Table 3:** Performance of gene feature selection methods with KNN classifier (high) and SVM classifier (low) in multiclass datasets

Dataset: Lymphoma								
	DKBCGS	GINI	Χ^2^-Statistic	Info.Gain	KW	RF	MRMR	KBCGS
ACC	**1.0000**	**1.0000**	**1.0000**	**1.0000**	0.8617	0.9756	**1.0000**	**1.0000**
Gene content	25	70	26	49	22	16	29	35
TPR	1.0000	1.0000	1.0000	1.0000	0.8617	0.9756	1.0000	1.0000
TIME(s)	**0.3412**	0.8944	2.3579	1.2561	7.4577	3.3144	1.5922	0.7541
Dataset: Lung cancer								
	DKBCGS	GINI	Χ^2^-Statistic	Info.Gain	KW	RF	MRMR	KBCGS
ACC	0.9554	0.9443	0.9499	**0.9641**	0.9273	0.9472	0.9291	0.9514
Gene content	32	82	97	65	39	88	50	40
TPR	0.9243	0.9033	0.9185	0.9012	0.9210	0.9123	0.9042	0.9155
TIME(s)	**0.1215**	0.2250	0.1954	0.1857	1.6478	0.5111	0.2931	0.3171
Dataset: Lymphoma								
	DKBCGS	GINI	Χ^2^-Statistic	Info.Gain	KW	RF	MRMR	KBCGS
ACC	**1.0000**	0.994	1.0000	1.0000	0.9283	0.9963	1.0000	1.0000
Gene content	35	34	34	16	28	17	27	40
TPR	1.0000	0.994	1.0000	1.0000	0.9283	0.9963	1.0000	1.0000
TIME(s)	**0.3412**	0.8944	2.3579	1.2561	7.4577	3.3144	1.5922	0.7541
Dataset: Lung cancer								
	DKBCGS	GINI	Χ^2^-Statistic	Info.Gain	KW	RF	MRMR	KBCGS
ACC	0.9151	0.9041	0.9115	**0.9229**	0.9102	0.9087	0.9199	0.9100
Gene content	64	87	75	89	71	60	77	74
TPR	0.9172	0.9005	0.9124	0.9285	0.9089	0.9114	0.9207	0.9122
TIME(s)	**0.5736**	1.8912	3.4551	2.4972	6.9322	4.1978	2.1207	1.0044

When using the lung cancer gene expression data, there is a substantial improvement in the accuracy of the classification using the double RBF-kernel algorithm for each of the feature subsets, which demonstrates that the double RBF-kernel method can select the appropriate genes efficiently compared to other methods. For lung cancers, the feature genes selected by the double RBF-kernel algorithm also result in a higher accuracy. It not only improves the accuracy of the classification of gene expression data, but also identifies informative genes that are responsible for causing diseases. Therefore, the double RBF-kernel method is better than the Χ^2^-Statistics, MRMR, Relief-F, Information Gain, and Kruskal-Wallis test. Also, the Information Gain method turns out to be highly competitive.

In the second part of the experiment, the expression level of the selected genes (dataset: Lymphoma) was represented as before in Fig. 8(a). It shows the expression profiles for the top-ranked genes selected by fusion in the form of the colormap. There is a visible border between the different groups. Note that the images of the expression profiles for 20 genes are chosen randomly, see Fig. 8(b). There is a significant difference between both images, which demonstrates the performance of the proposed selection procedure.

### Differential gene expression analysis

**Fig. 9:**
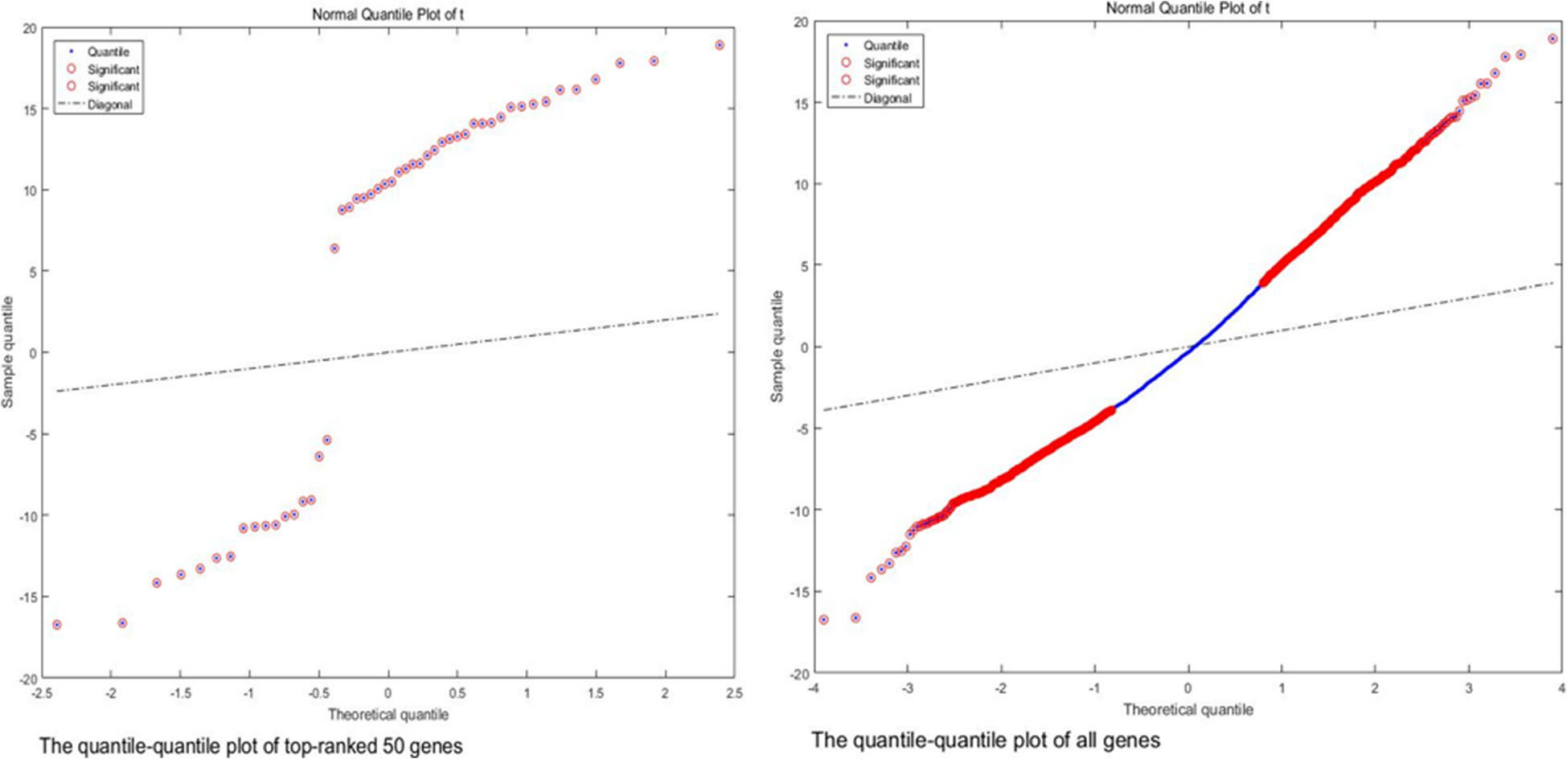
The quantile-quantile plot of top ranked 50 genes (left) and all genes (right). The horizontal axis is the theoretical quantile, the vertical axis is the sample quantile, and the red circle represents a significant gene

**Fig. 10:**
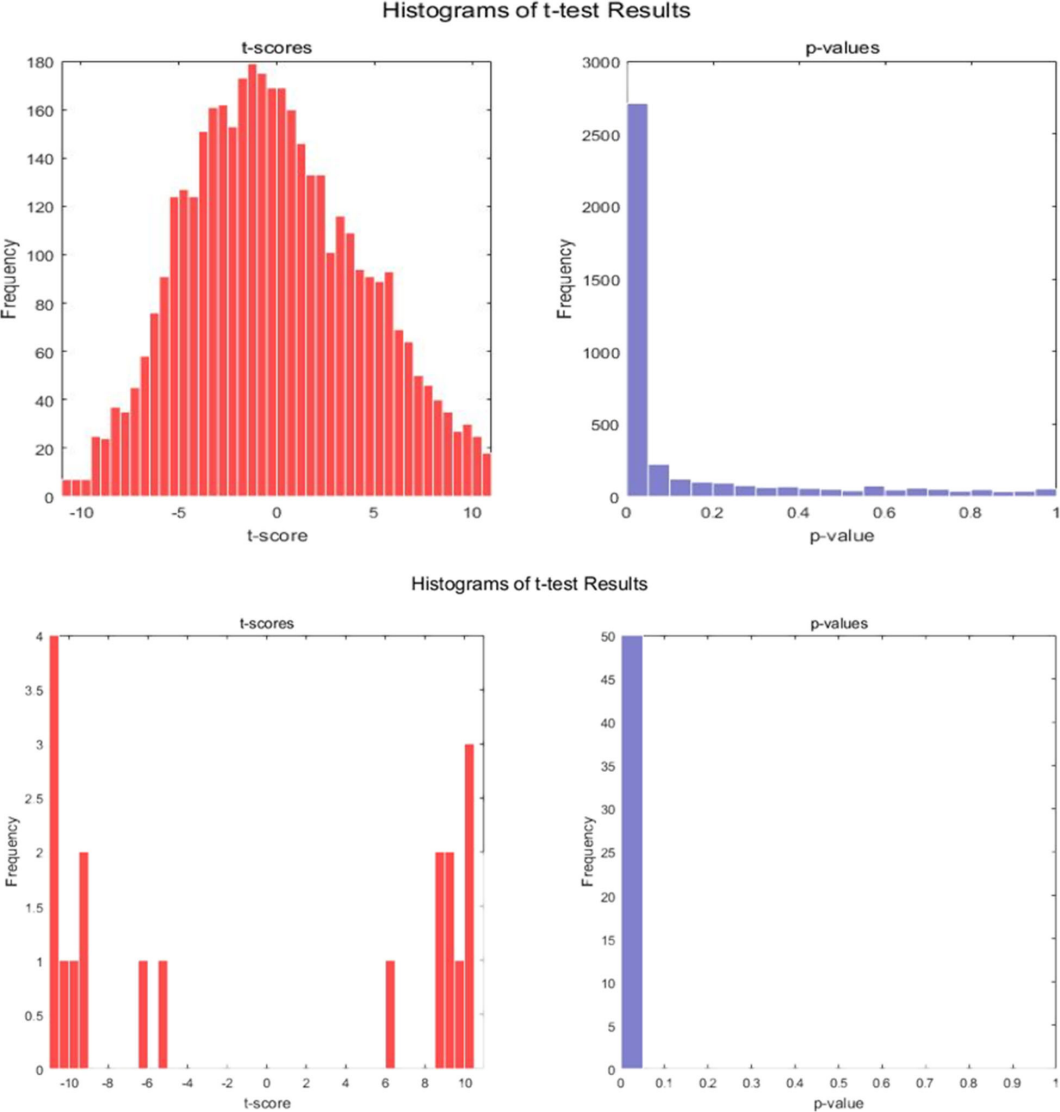
The t-score plot and the *p*-value plot of the top ranked 50 genes (bottom 2 graphs) and all genes (upper 2 graphs). The horizontal axis is the t-score/p-value, the vertical axis is the number of genes in each interval

The top 50 genes of Gastric cancer dataset were analyzed by applying the paired *t*-test method to obtain the t-score, *p*-value plot and the quantile-quantile plot of these genes. The quantile-quantile plot is mainly for identifying the gene expression levels of two classes. The results, as shown in Figs. 9 and 10, clearly show the difference between the feature genes obtained by DKBCGS and the original data. All the genes were divided into genes with significant attributes, and have a low *p-*value (average *p*-value = 0.023). Finally, this proves that DKBCGS has a certain statistical significance.

The t-score plot shows the normality of the data and the rationality of using the paired t-test. We can also conclude from the histogram of p-value that the paired t-test is significant because of the vast majority of p-value falls in the very end of the group of the histogram.

Between two groups of variables, a t-test is performed on each gene to identify significant differences in all genes and feature genes selected by our method, and a normal quantile map can be obtained by t-scores. A histogram of t-scores and *p*-values was used to study the test results.

## Conclusion

The number choice of kernels could typically depend on the level of heterogeneity of the datasets. Experiments on gene expression datasets show that double RBF-kernel outperforms all other used feature selection methods in terms of classification accuracies for both two-class datasets and multiclass datasets, especially in those datasets with small samples. The performances of double RBF-kernel learning in classification make it well suited alternatives to one RBF-kernel learning.

The use of known performance measures, such as accuracy, TNR, and TPR, clearly showed the high potential of the proposed method for performing classification tasks in bioinformatics and related disciplines. The initial value of δ as a ranking criterion was a key issue here for performing feature gene selection. In this paper, a flexible model for cancer gene expression classification and feature gene selection was proposed, which can adjust the parameters when using different datasets through cross validation to achieve the best result. The performance of the proposed method was compared to six classical methods, demonstrating that it could outperform existing methods in the identification of feature cancer genes. In conclusion, the proposed method is superior in accuracy and run-time for both two-class datasets and multiclass datasets, especially for those datasets with small samples. Furthermore, the results show that our method is computationally efficient. Also, the double-kernel learning may not be good at handling a super large scale of data. Future work could investigate computational aspects more in-depth on a large scale and use graph-based kernels to process gene networks.

## Additional files

Additional file1: Existing gene selection methods: a brief introduction. (DOCX 18 kb)

Additional file 2: SVM and KNN classifiers. (DOCX 18 kb)

Additional file 3: Dataset descriptions. (DOCX 16 kb)

Additional file 4: Further comparison for other datasets**. Table S1.** Average performance in KNN and SVM classifiers of DKBCGS and KBCGS (two classification). **Table S2.** Average performance in KNN and SVM classifiers of our method and KBCGS (multi-classification). **Table S3.** Performance of gene feature selection methods with KNN classifier (high) and SVM classifier (low) in two-class datasets. (DOCX 27 kb)

Additional file 5: top-50 gene expression**. Figure S1.** First fifty top-ranked gene expression level by different methods. The horizontal axis is the number of characteristic genes, the vertical axis is the gene expression level, and the black line represents the mean gene expression difference between the normal sample and the cancer sample. (PNG 596 kb)

## Abbreviations

ACC: Accuracy
DCBL: Diffuse large B-cell lymphoma
GRBF: Gaussian radial basis function
KNN: K-Nearest Neighbor
MRMR: Maximum relevance and minimum redundancy
PCA: Principal component analysis
POLY: Polynomial kernel function
RBF: Radial Basis Function
SRBCTs: Small round blue cell tumors
SVM: Support Vector Machine
TNR: True negative rate
TPR: True positive rate
