# Supplementary material for: Feature selection of gene expression data for Cancer classification using double RBF-kernels
Source: BMC Bioinformatics. 2018 Oct 29;19:396. doi: 10.1186/s12859-018-2400-2 (PMC6206917; doi:10.1186/s12859-018-2400-2)
Supplement: Supplementary file 1 — Existing gene selection methods: a brief introduction. (DOCX 18 kb) [file 12859_2018_2400_MOESM1_ESM.docx]

Additional file1. Existing gene selection methods: a brief introduction

### Relief-F

The relief algorithm was first proposed by Kira [13], initially confined for the classification of two types of data. The relief-F algorithm is in fact an extension of the Relief algorithm, which can deal with noisy, incomplete and multiclass datasets. In general, a random sample instance U is generated from m instances and the relevance values are updated based on the difference between the selected instance U and the nearest instances of the same H (called nearest hit) and different class M(C) (called nearest miss of class C). It gives more weight to features that discriminate the instance from neighbors of different classes. The weights are updated by considering the average contribution of nearest misses M(C). The average contribution also takes prior probability of each class into account. The weight of i th feature Xi is given by:

$w=w_{i}-\frac{\Psi\left（ X_{I},U,H \right）}{m}+\sum_{C\notin C_{R}} \frac{P(C)\times\Psi\left（ X_{I},U,M(C) \right）}{m}$ , (S1)

where$, \Psi\left（ X_{I},U,H \right）$ represents the distance function between the sample instance U and the nearest hit H or the nearest miss M(C).

### Information Gain

The information gain has been widely used as an attribute selection method for generating and analyzing Decision Trees [14]. For each feature Xi, the information gain is measured by:

$\mathrm{InfoGain}\left( X_{i} \right)=H\left( C \right)-H(C/X_{i})$, (S2)

where, $H\left( C \right)=-\sum_{c\in C} p(c)\log_{c} p(c)$

and $H\left( \frac{C}{X_{i}} \right)=-\sum_{x\in X_{i}} p(c/x)\sum_{c\in C} p(c/x)\log_{c} p(c/x),$

where, C = {Cj} is the class set j = 1, 2, . . ., l. The entropy-based discretization method is generally used for analyzing gene expression data.

### MRMR

MRMR (minimum redundancy-maximum relevance) was proposed by Ding and Peng [15]. It selects features by minimizing redundancy among them with maximal relevance based on information theory. The maximum relevance criterion is to find a set of S that satisfies the following formula from all possible sets containing *m* features:

$S_{m}=argmax(\frac{1}{|S|}\sum_{X_{i}\in S} I(X_{i};C))$ (S3)

It can be seen from this formula that the maximum correlation criterion aims to find the set of S*m* to meet that the mutual information of each feature in S*m* and the target variable C of the sum of the average will be maximized. The minimum redundancy criterion is to find a S*m* from all possible sets of *m* eigenvectors, and the similarity between each feature variable of the set is minimal.

$S_{m}=argmin(\frac{1}{|S|^{2}}\sum_{X_{i},X_{j}\in S} I(X_{i};X_{j}))$ (S4)
