# Supplementary material for: Feature selection of gene expression data for Cancer classification using double RBF-kernels
Source: BMC Bioinformatics. 2018 Oct 29;19:396. doi: 10.1186/s12859-018-2400-2 (PMC6206917; doi:10.1186/s12859-018-2400-2)
Supplement: Supplementary file 2 — SVM and KNN classifiers. (DOCX 18 kb) [file 12859_2018_2400_MOESM2_ESM.docx]

## Additional file 2. SVM and KNN classifiers

Support vector machine (SVM) is a general machine learning method [13]. For a two-class classification problem, we assume the training set T={(x_1_, y_1_), (x_2_, y_2_),…, (x_n_, y_n_)}, where $x\in\chi=R^{n} i$s the sample vector, $y_{i}\in Y=\{+1,-1\}$ is the label, n is the number of samples. Assume there is a mapping$\varphi(x_{i})$, which maps the data xi from the original vector space$\chi$ to the high-dimensional feature space. By introducing the slack variable$\xi_{i}$, the original problem of SVM can be expressed as: $\min_{\alpha} \frac{1}{2}|\left| w \right||^{2}+C\sum_{i=1}^{n} \xi_{i}$

$s.t.\left\{ \begin{aligned} y_{i}\left( w\cdot\varphi\left( x_{i} \right)+b \right)\geq1-\xi_{i} \\ \xi_{i}\geq0 \end{aligned} ; \right. i=1,2,\ldots,n$ (S5)

The Lagrange function method can be derived by:

$$\max_{\alpha} -\frac{1}{2}\sum_{j=1}^{n} \sum_{i=1}^{n} \alpha_{i}\alpha_{j}y_{i}y_{j}k\left( x_{i},x_{j} \right)+\sum_{i=1}^{n} \alpha_{i}$$

$s.t. \left\{ \begin{aligned} \sum_{i=1}^{n} \alpha_{i}y_{i}=0 \\ 0\leq\alpha_{i}\leq C \end{aligned};i=1,2,\ldots,n \right.$ (S6)

$k\left( x_{i},x_{j} \right)$ is the positive definite kernel function that satisfies the Mercer theorem and C is the penalty parameter.

The KNN algorithm has been widely used due to its low classification error rate. We assume an unknown sample set U = {u1, u1,...,um} and the training set T={(x_1_, y_1_), (x_2_, y_2_),…, (x_n_, y_n_)}, where $x\in\chi=R^{n}$ is the sample vector,$\mathrm{and}y_{i}\in Y$ is the label.

As to KNN classifier, we use the Euclidean distance as a distance function and set K=5 due to the performance in the validation set:

$D\left( x_{i},x_{j} \right)=\sqrt{\sum(x_{i}-x_{j})^{2}}$ (S7)
