# Supplementary material for: Feature selection of gene expression data for Cancer classification using double RBF-kernels
Source: BMC Bioinformatics. 2018 Oct 29;19:396. doi: 10.1186/s12859-018-2400-2 (PMC6206917; doi:10.1186/s12859-018-2400-2)
Supplement: Supplementary file 3 — Dataset descriptions. (DOCX 16 kb) [file 12859_2018_2400_MOESM3_ESM.docx]

## Additional file 3. Dataset descriptions

### Diffuse large B-cell lymphoma

The diffuse large B-cell lymphoma (DLBCL) dataset [16]**.** consists of two types of 77 samples, i.e., 58 samples of DLBCL and 19 samples of FL (follicular lymphoma). There are 6,817 genes in the dataset, which is available on the website: www.genome.wi.mit.edu/MPR/lymphoma/.

### Gastric cancer

The gastric cancer dataset [17]. consists of 40 samples, half from gastric cancer patients and half from normal patients. The size of the dataset is 1,519 and it is available on the website: http:// www.codebus.net/d-H9pw.html.

### Multi-Cancer

The multi-cancer dataset [18]. consists of 152 samples from five different types. The expression profiles of cancer and adjacent normal tissues from 76 patients (20 with gastric cancer, 20 with colon cancer, 16 with liver cancer and 20 with lung cancer) were studied using microarray data and a set of lncRNAs as well as PCGs were identified as potential biomarkers, see https://www.ncbi.nlm.nih.gov/geo/query/acc.cgi?acc=GSE70880.

### Lymphoma

The lymphoma dataset [1]. consists of 40 samples, which were obtained from sick and normal patients, DLBCL, FL and chronic lymphocytic leukemia (CLL). There are 4026 genes in the dataset, see https://llmpp.nih.gov/lymphoma/.
