# Supplementary material for: Feature selection of gene expression data for Cancer classification using double RBF-kernels
Source: BMC Bioinformatics. 2018 Oct 29;19:396. doi: 10.1186/s12859-018-2400-2 (PMC6206917; doi:10.1186/s12859-018-2400-2)
Supplement: Supplementary file 4 — Further comparison for other datasets. Table S1. Average performance in KNN and SVM classifiers of DKBCGS and KBCGS (two classification). Table S2. Average performance in KNN and SVM classifiers of our method and KBCGS (multi-classification). Table S3. Performance of gene feature selection methods with KNN classifier (high) and SVM classifier (low) in two-class datasets. (DOCX 27 kb) [file 12859_2018_2400_MOESM4_ESM.docx]

## Additional file 4. Further comparison for other datasets

Table S1. Average performance in KNN and SVM classifiers of DKBCGS and KBCGS (two classification).

| DATASET | Method | Acc | Sn | Sp | Time |
| --- | --- | --- | --- | --- | --- |
| DLBCL | DKBCGS | 0.9874 | 0.9975 | 0.9762 | 0.0874 |
|  | KBCGS | 0.9845 | 0.9579 | 0.9931 | 0.2148 |
| AML_ALL | DKBCGS | 0.9975 | 1 | 0.9950 | 0.0916 |
|  | KBCGS | 0.9754 | 1 | 0.9280 | 0.2930 |
| Lung cancer | DKBCGS | 0.9211 | 0.9342 | 0.9015 | 0.0125 |
|  | KBCGS | 0.8700 | 0.8750 | 0.8760 | 0.0370 |
| Prostate cancer | DKBCGS | 0.9624 | 0.9516 | 0.9744 | 0.1533 |
|  | KBCGS | 0.9517 | 0.9231 | 0.9780 | 0.4503 |

Table S2. Average performance in KNN and SVM classifiers of our method and KBCGS (multi-classification).

| DATASET | Method | Acc | TPR | Time |
| --- | --- | --- | --- | --- |
| Brain_Tumor | DKBCGS | 0.9237 | 0.9105 | 0.1262 |
|  | KBCGS | 0.9067 | 0.8033 | 0.3118 |
| Lymphoma | DKBCGS | 1 | 1 | 0.0841 |
|  | KBCGS | 1 | 1 | 0.2292 |
| NCI60 | DKBCGS | 0.8057 | 0.7915 | 0.1744 |
|  | KBCGS | 0.7824 | 0.7503 | 0.2004 |
| SRBCT | DKBCGS | 1 | 1 | 0.0711 |
|  | KBCGS | 1 | 1 | 0.0768 |

Table S3. Performance of gene feature selection methods with KNN classifier (high) and SVM classifier (low) in two-class datasets

| Dataset: Gastric cancer | | | | | | | | |  |
| --- | --- | --- | --- | --- | --- | --- | --- | --- | --- |
|  | DKBCGS | GINI | χ^2^-Statistic | Info.Gain | KW | RF | MRMR | KBCGS | |
| TP | 0.9902 | 0.9664 | 0.9875 | 0.9834 | 0.9322 | 0.9548 | 1 | 0.9716 | |
| FP | 0 | 0.1274 | 0.9367 | 0 | 0.1543 | 0.0244 | 0 | 0.0463 | |
| TN | 1 | 0.9677 | 0.9969 | 1 | 0.8915 | 0.9498 | 1 | 0.9826 | |
| FN | 0.0121 | 0.0934 | 0.0032 | 0.1003 | 0.1465 | 0.1548 | 0 | 0.0732 | |
| Dataset: DLBCL | | | | | | | | |  |
|  | DKBCGS | GINI | χ^2^-Statistic | Info.Gain | KW | RF | MRMR | KBCGS | |
| TP | 0.9964 | 0.9123 | 0.9466 | 0.9644 | 0.9365 | 0.9414 | 0.9755 | 0.9783 | |
| FN | 0 | 0.0012 | 0.2589 | 0.1753 | 0.1324 | 0.0327 | 0.0487 | 0.0254 | |
| TN | 0.9933 | 0.9645 | 0.9564 | 0.9355 | 0.9677 | 0.9517 | 0.9743 | 0.9785 | |
| FN | 0.0023 | 0.0534 | 0.0648 | 0.1865 | 0.1064 | 0.1004 | 0.0438 | 0.0173 | |

| Dataset: Gastric cancer | | | | | | | | |  |
| --- | --- | --- | --- | --- | --- | --- | --- | --- | --- |
|  | DKBCGS | GINI | χ^2^-Statistic | Info.Gain | KW | RF | MRMR | KBCGS | |
| TP | 1 | 0.9743 | 0.9912 | 0.9612 | 0.9213 | 0.9544 | 1 | 0.9923 | |
| FP | 0 | 0.0011 | 0.0435 | 0.0121 | 0.0224 | 0.0544 | 0.0003 | 0.0105 | |
| TN | 1 | 0.9765 | 0.9812 | 0.9313 | 0.9566 | 0.9464 | 0.9954 | 0.9886 | |
| FN | 0 | 0.0023 | 0.0654 | 0.0145 | 0.0332 | 0.0125 | 0.0543 | 0.0631 | |
| Dataset: DLBCL | | | | | | | | |  |
|  | DKBCGS | GINI | χ^2^-Statistic | Info.Gain | KW | RF | MRMR | KBCGS | |
| TP | 1 | 1 | 1 | 1 | 1 | 0.9766 | 0.9877 | 0.9976 | |
| FP | 0 | 0 | 0 | 0 | 0.0016 | 0.0012 | 0.1001 | 0.0453 | |
| TN | 1 | 1 | 1 | 1 | 0.9213 | 0.9752 | 1 | 0.9944 | |
| FN | 0 | 0 | 0 | 0 | 0.1274 | 0.0984 | 0.0043 | 0.0012 | |
