# Supplementary figures and images for: Feature selection of gene expression data for Cancer classification using double RBF-kernels
Source: BMC Bioinformatics. 2018 Oct 29;19:396. doi: 10.1186/s12859-018-2400-2 (PMC6206917; doi:10.1186/s12859-018-2400-2)

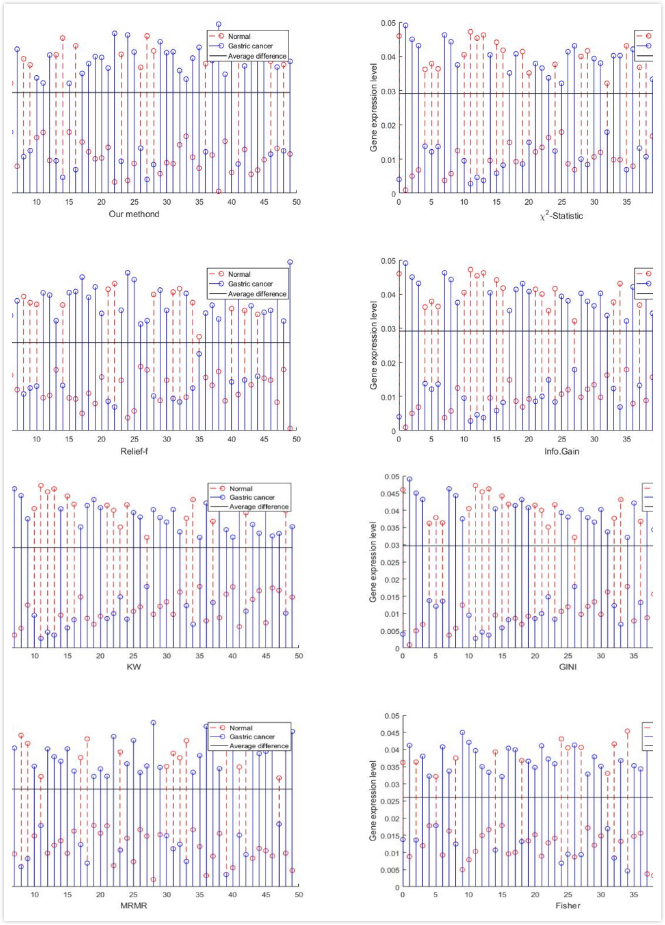

Supplement: Supplementary file 5 — top-50 gene expression. Figure S1. First fifty top-ranked gene expression level by different methods. The horizontal axis is the number of characteristic genes, the vertical axis is the gene expression level, and the black line represents the mean gene expression difference between the normal sample and the cancer sample. (PNG 596 kb) [file 12859_2018_2400_MOESM5_ESM.png]
